# Prediction of daily milk production from the linear body and udder morphometry in Holstein Friesian dairy cows

**DOI:** 10.14202/vetworld.2020.471-477

**Published:** 2020-03-13

**Authors:** Soeharsono Soeharsono, Sri Mulyati, Suzanita Utama, Wurlina Wurlina, Pudji Srianto, Tjuk Imam Restiadi, Imam Mustofa

**Affiliations:** 1 Department of Veterinary Anatomy, Faculty of Veterinary Medicine, Universitas Airlangga, Kampus C Unair, Jl. Mulyorejo, Surabaya-60115, Indonesia; 2 Department of Veterinary Reproduction, Faculty of Veterinary Medicine, Universitas Airlangga, Kampus C Unair, Jl. Mulyorejo, Surabaya-60115, Indonesia

**Keywords:** body length, front udder height, milk production, rear udder height, udder circumference

## Abstract

**Aim:**

This study aimed to develop equations to predict daily milk production (DMP) based on linear body and udder morphometry of Holstein Friesian (HF) dairy cows.

**Materials and Methods:**

The experiment was conducted on 174 lactating HF dairy cows reared by farmers at different locations under similar conditions. The age, parity, and body condition score of experimental animals were limited to 0.25 of the standard deviation value above or below the average. The average DMP was based on farmers’ records. Morphometry components, i.e., body length (BL); chest circumference (CC); front udder height (FUH), rear udder height (RUH); and udder circumference (UC) were directly measured using a tape; meanwhile, body weight (BW) was estimated using the Indonesia Winter formula. The relationship variables of morphometry components (body and udder morphometry) and BW on DMP were analyzed by regression.

**Results:**

The result showed no correlation (p>0.05) between CC and BW on DMP. Meanwhile, DMP obtained linear regression (p<0.05) with the mathematical equation: 1.30+0.11*BL; 13.90+0.41*FUH; 11.02+0.18*RUH; and 3.87+0.16*UC.

**Conclusion:**

This study shows that the DMP of dairy cows could be predicted based on their BL and udder morphometry.

## Introduction

Holstein Friesian (HF) cows have been reared in Indonesia since the 18^th^ century. The cows have to adapt to highlands of up to 700 m and lowlands that are 0-100 m above sea level with temperatures and humidity of 16-23°C and 92% or 28–35°C and 54%, respectively [1]. The weather influences feed intake and the efficiency of milk production. Over the centuries, this has led to physiological and morphological adaptations. Under high ambient temperatures, livestock reduces dry matter intake to decrease their metabolic heat production; meanwhile, for high-yielding dairy cows, a high intake of dry matter is needed [2]. The difference in body weight (BW) is, of course, followed by changes in body morphometry and milk production. The average BW of a cow reared in sub-tropical areas is higher than that of a cow reared in tropical areas, which affects average milk production [3]. Milk is synthesized in the milk-producing unit called alveoli in the mammary gland. The capacity for milk synthesis depends on the number and efficiency of functional mammary epithelial cells. It is affected by protein intake [4], hypothetically influenced by body and udder morphometry.

The problem faced by smallholder farmers is having limited facilities to find out the BW and production records of cows. Farmers choose a dairy cow based only on the visual estimation of body and udder sizes without any quantitative measurements. This method is certainly far from accurate to get dairy cows with high milk production. For this reason, there is a necessity for a simple measurement method that is cheap and can be done by everyone as a guideline for estimating a productive dairy cow. The principle of the estimation method is to transform a known variable to be the desired variable value. The relationship of the body size to other parameters can be modeled in the regression equation [5]. BWs can be predicted through body morphometry based on body length (BL) and chest circumference (CC) [6] using the Indonesia Winter formula. Measurements using the estimation method, although not more accurate than direct measurements, have significant uses in terms of practicality, especially at the level of the smallholder farmers.This study aimed to develop equations to predict daily milk production (DMP) based on linear body and udder morphometry of HF dairy cows.

## Materials and Methods

### Ethical approval

This research was conducted under the supervision of an assessor of the Animal Care and Use Committee (ACUC) from the Faculty of Veterinary Medicine, Universitas Airlangga, Surabaya, Indonesia.

### The location of the study and study period

This study was conducted in Jambuwer Village, Wagir District, Malang Regency, Indonesia during eight months (September 2018 - April 2019). The geographic location and the data of the weather were obtained from the global positioning system [7] and Meteorological, Climatological, and Geophysical Agency [8], respectively. Wagir District is located ±450 m above the sea level (ASL) at the slopes of Mount Kawi, with temperatures ranging from 11 to 25°C, with an average humidity of 79%-86%. Geographically, it was located at 112°17’10”-112°57’00” east longitude (EL) and 7°44’55.11”-8°26’35.45” south latitude (SL). Jambuwer Village was in the Wagir District, on 433 ASL, geographically located at 112°31’3” EL and 8°1’31” SL, with temperature ranges from 18 to 28°C and average humidity of 86%.

The mathematical equation of linear regression was then cross-checked on cows at two different locations. The first was Precet Village, Wagir District, located 474 m ASL, at 112°30’36” EL and 8°0’8” SL, with an ambient temperature of 18-28° C and average humidity of 74%. The second was Geger Village, Sendang District, located 652 m ASL, at 111°50’0” EL, and 7°57’0” SL, with an ambient temperature of 18-27°C and average humidity of 88%.

### Experimental animals

This study used HF cows owned by smallholder farmers. The cows were healthy and lactating. The age, parity, and body condition score (BCS) of experimental animals were limited to 0.25 of the standard deviation value above or below the average. The HF cows were aged 4-8 years, had a parity of 2-5, and BCS of 4-6 on a nine-point scale. The cows were randomly selected from the population based on these criteria.

### Management of animals

The cows were cowshed all day long and managed traditionally. Milking was conducted twice a day, in the morning, at 03.00-06.00, and in the afternoon, at 13.00-15.00. In the early morning, the farmers prepared for milking by cleaning the pen, feeding the cows concentrate, and washing them. Milking was conducted by hand, one cow at a time. The milk yield of each cow was measured, and the milk collected in the milk can then delivered to the milk collecting point of the cooperative. In about 1 h after milking, the cows were fed grass.

Dairy cows were fed 25-35 kg of elephant grass and 9-16 kg of concentrate daily, while water was available *ad libitum*. In the morning, the feed given was as much as 40%, while in the afternoon, it was 60%. The farmers obtained elephant grass from fields around the cowshed for a 1-day stock only. Meanwhile, a concentrate containing 16-18% rough protein was supplied by the cooperative.

### Recording of parameters

The measurements of body morphometry, i.e., BL and CC, and udder morphometry, i.e., front udder height (FUH), rear udder height (RUH), and udder circumference (UC), were carried out using a measuring tape scale of 200 cm. The BL was measured from the manubrium of the sternum to the tip of the tuber ischia, while the value of the CC was obtained by looping the measuring tape on the body of the cow behind the scapula. The parameter measurements of each cow were obtained twice a day before milking for three replications at 7-day intervals. Based on the BL (cm) and CC (cm) of each cow, the BW (kg) of the cow could be calculated based on the Winter Indonesia formula: BW=(CC)^2^×BL)/10,815.15 [9]. The FUH was measured perpendicularly from the point of attachment in the ventral abdomen to the lowest point of the front udder; RUH was straight upright from the highest mammary point at the cow’s caudal body to the lowest point of the rear udder, and UC was measured based on the largest UC between the two hind legs of the cow. The DMP was the average daily milk yield of the last lactation period collected from the farmer record.

### Statistical analysis

All the variables were analyzed to find out the normality of the distribution using the Kolmogorov–Smirnov test, followed by the correlation of one to each other. When the correlation among variables was significant, the study would continue to find the linear regression to the average DMP (l/day). A linear regression line has the equation: Y=a+bX, where “X” is the explanatory or the independent variable, and “Y” is the dependent one. The slope or the beta coefficient of the line is “b”, while “a” is the intercept (the value of y when x=0). The value of coefficient correlation (r) and its meaning, i.e., 0.00-0.19 is very weak; 0.20-0.39 is weak, 0.40-0.59 is moderate, 0.60-0.79 is strong, and 0.80-1.0 is very strong [10]. If regression is met, the trailing checks of goodness of fit include the R-squared and hypothesis testing of individual parameters by t-tests. The coefficient of determination, R^2^, represents the proportion (%) of the variance for a dependent variable that is explained by an independent variable [11]. The computed equation of linear regression cross-checked the suitability between the predicted DMP (e-DMP) then compared it to real DMP (r-DMP) in other locations of smallholder dairy farmers. Statistical analysis was conducted at a 95% level of significance.

## Results

Based on the criteria mentioned earlier, 174 cattle were obtained that meet the requirements. Homogeneity examination using a Kolmogorov–Smirnov test of all the parameters showed that the data were normally distributed (p>0.05). The average age, parity, and BCS of the cows were 4.87±1.13 years, 2.23±0.12, and 5.14±0.15, respectively. The daily average of cows feeding was 33.91±0.73 kg elephant grass and 10.44±0.64 kg concentrate (22.77±1.17% of feed); meanwhile, the average DMP was 17.66±3.47 l/d. The average body morphometry: BL was C166.68±7.28 cm, CC was 188.79±8.46 cm, and BW was 550.57±60.98 kg. Furthermore, the udder morphometry: FUH was 9.16±1.16 cm, RUH was 37.81±7.76 cm, and UC was 87.86±15.33 cm (Table-1).

**Table-1:** Age, parity, BCS, nutrient intake, daily milk production, estimated body weight based on the Winter formula, body length, chest circumference, front udder height, rear udder height, and udder circumference of Friesian Holstein dairy cows (n=174).

Variables	Minimum	Maximum	Mean	Standard deviation
Age (years)	4	8	4.87	1.13
Parity	2	5	2.23	0.12
Body condition score (BCS, nine scales)	4	6	5.14	0.15
Nutrient intake:				
Forage (kg)	25	35	33.91	0.73
Concentrate (kg)	9	16	10.44	0.64
% Concentrate	16.57	31.38	22.77	1.17
Daily milk production (DMP, l/day)	7	37	17.66	3.47
Body length (BL, cm)	153	179	166.68	7.28
Chest circumference (CC, cm)	168	214	188.79	8.46
Estimation of bodyweight (BW, kg)	411.38	743.55	550.57	60.98
Front udder height (FUH, cm)	7	17	9.16	1.16
Rear udder height (RUH, cm)	18	56	37.81	7.76
Udder circumference (UC, cm)	51	160	87.86	15.33

### Correlation among variables

There was significant correlation (p<0.05) between BL with CC, BW, FUH, RUH, and DMP; CC with BL and BW; BW with BL and CC; FUH with BL, RUH, UC, and DMP; RUH with BL, BW, FUH, UC, and DMP; UC with FUH, RUH, and DMP; and DMP with BL, FUH, RUH, and UC (Tables-2 and 3).

**Table-2:** Pearson matrix correlation among variables.

Variables	BL	CC	BW	FUH	RUH	UC	DMP
BL	**1**	**0.2999**	**0.6364**	**0.2124**	**0.2202**	0.0855	**0.1520**
CC	**0.2999**	**1**	**0.9251**	0.0771	0.1332	0.0202	0.0596
BW	**0.6364**	**0.9251**	**1**	0.1451	**0.1886**	0.0511	0.1109
FUH	**0.2124**	0.0771	0.1451	**1**	**0.8611**	**0.3317**	**0.1623**
RUH	**0.2202**	0.1332	**0.1886**	**0.8611**	**1**	**0.4205**	**0.2481**
UC	0.0855	0.0202	0.0511	**0.3317**	**0.4205**	**1**	**0.4370**
DMP	**0.1520**	0.0596	0.1109	**0.1623**	**0.2481**	**0.4370**	**1**

Values in bold were different from 0 with a significance level of <0.05. DMP=Daily milk production, BL=Body length, CC=Chest circumference, BW=Bodyweight, FUH=Front udder height, RUH=Rear udder height, UC=Udder circumference

**Table-3:** Level significance of correlation among variables.

Variables	BL	CC	BW	FUH	RUH	UC	DMP
BL	**0**	**0.0001**	**0.0000**	**0.0049**	0.0485	0.2619	**0.0452**
CC	**<0.0001**	**0**	**<0.0001**	0.3122	0.0177	0.7911	0.4346
BW	**<0.0001**	**<0.0001**	**0**	0.0561	0.0356	0.5032	0.1451
FUH	**0.0049**	0.3122	0.0561	**0**	0.7415	**<0.0001**	**0.0324**
RUH	**0.0485**	0.0177	**0.0356**	**0.7415**	**0**	**0.1768**	**0.0010**
UC	0.2619	0.7911	0.5032	**<0.0001**	0.1768	**0**	**<0.0001**
DMP	**0.0452**	0.4346	0.1451	**0.0324**	**0.0010**	**<0.0001**	**0**

Values in bold were different from 0 with a significance level <0.05. DMP=Daily milk production, BL=Body length, CC=Chest circumference, BW=Bodyweight, FUH=Front udder height, RUH=Rear udder height, UC=Udder circumference

### The effect of morphometry on DMP

**Figure-1:**
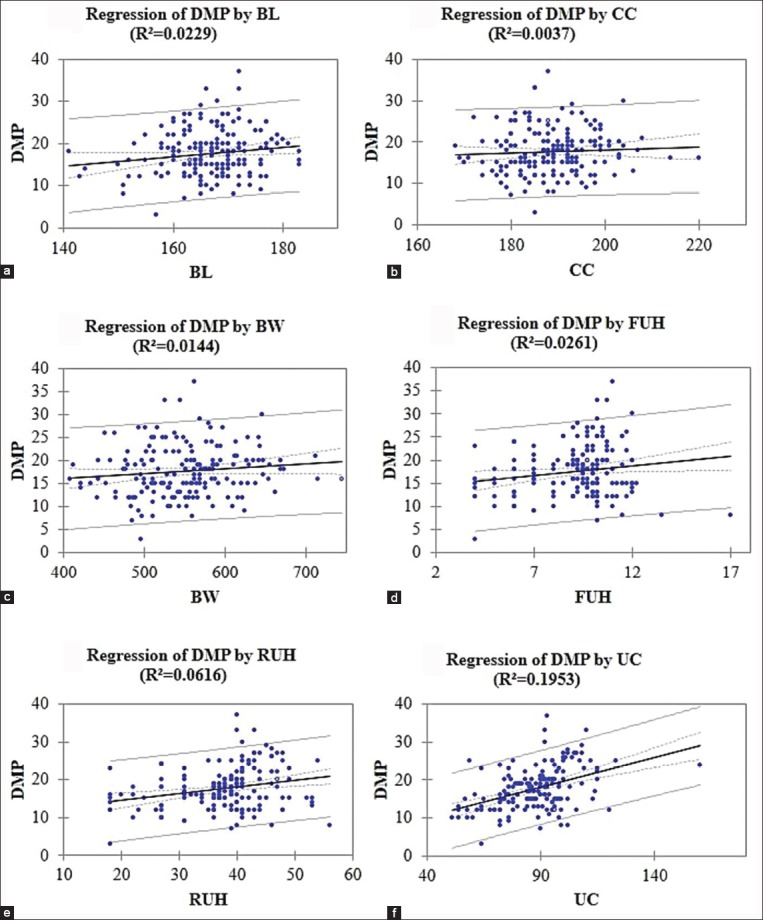
Regression equation of daily milk production (l/d) based on body length (a), chest circumference (b), bodyweight (c), front udder height (d), rear udder height (e), and udder circumference (f) in cm.

In this study, the e-DMP was based on the body components that were BL, CC, BW, FUH, RUH, and UC. Four of them were significant correlations (p<0.05) to DMP, i.e., BL, FUH, RUH, and UC (Tables-2 and 3). Mathematical equation and regression graph of the relationship of body components to DMP are presented in Table-4 and Figure-1, respectively.

**Table-4:** Linear regression of morphometry of cows on DMP.

Equation	p-value	R^2^(%)
DMP=−1.30+0.11*BL	**0.045**	**2.31**
DMP=10.25+0.04*CC	0.428	0.36
DMP=11.56+0.01*BW	0.115	1,23
DMP=13.90+0.41*FUH	**0.033**	**2.63**
DMP=11.02+0.18*RUH	**0.0010**	**6.16**
DMP=3.87+0.16*UC	**<0.0001**	**19.53**

Values in bold were different from 0 with a significance level <0.05. DMP=Daily milk production, BL=Body length, CC=Chest circumference, BW=Bodyweight, FUH=Front udder height, RUH=Rear udder height, UC=Udder circumference

Based on the mathematical equation, Table-4 shows the e-DMP compared to actual DMP (r-DMP) in dairy cows in this study and of other locations (Table-5). The result showed that FUH and UC at location II were lower (p<0.05) compared to the other locations, followed by lower r-DMP, e-DMP-FUH, and e-DMP-UC. In contrast, the other predictors (BL and RUH) did not differ significantly (p> 0.05), both r-DMP and e-DMP.

**Table-5:** Crosscheck of the e-DMP compared to the r-DMP in a different location.

Variables	Location I (n=174)	Location II (n=103)	Location III (n=90)
BL	167.64±5.01	164.85±10.10	167.30±5.07
FUH	10.15±0.95^a)^	7.37±2.62^b)^	10.83±3.52^a)^
RUH	40.65±3.73	37.35±2.56	40.80±3.49
UC	93.97±10.75^a)^	77.92±9.03^b)^	95.90±11.57^a)^
r-DMP	18.94±5.57^a)^	15.23±2.39^b)^	19.27±6.58^a)^
e-DMP-BL	17.14±0.55	16.83±1.11	17.10±0.56
e-DMP-FUH	18.06±0.39^a)^	16.92±1.07^b)^	18.34±1.44^a)^
e-DMP-RUH	18.34±0.67	17.74±0.46	18.36±0.63
e-DMP-UC	18.91±1.72^a)^	16.34±0.53^b)^	19.21±1.85^a)^

BL=Body length, FUH=Front udder height, RUH=Rear udder height UC=Udder circumference, r-DMP=Real day milk production, e-DMP-BL=Predicted daily milk production based on body length, e-DMP-UH=Predicted daily milk production based on UH, e-DMP-UC=Predicted daily milk production based on udder circumference. The location I: Jambuwer Village (the location of the study) II: Geger Village, District of Tulungagung, III: Precet Village, District of Malang. Superscripts a and b in bold numbers were significantly different (p<0.05) in the same row. Real daily milk production

## Discussion

In this study, data on age, parity, BCS, and nutrient intake (Table-1) were statistically proven homogenous so that the DMP variability could be predicted based on body and udder morphometry. Morphometry is a variable of the breed that describes the capacity of the biological processes affecting milk production [12], and a phenotypic trait, which is a genetic expression in response to the environment, including nutrition. This study showed that there are four variables, i.e., BL, FUH, RUH, and UC that affect DMP significantly (p<0.05), so they could be used as estimators of DMP (Tables-2-4).

### The effect of body morphometry and BW on DMP

When the cow reaches 18 months of age, BL will not increase significantly anymore [13]. BL indirectly measures the vertebrae along with the tissue between the vertebrae arranged in a longitudinal line; meanwhile, CC not only measures the circumference of the bones forming the chest cavity but also the tissue attached, especially the muscles and skin. In the estimation of BW, the highest correlation is also determined by CC. Likewise, morphometric measurements to determine the right space amount in the dairy cowshed are also determined by the parameters of the thorax area [14]. In this study, there was no linear correlation between the BW and CC of dairy cows, on the one hand, and DMP, on the other hand. This fact is consistent with earlier reports that a decrease in BW is related to a reduction in the ratio of fat to protein; nevertheless, it does not affect milk production [15,16]. However, fluctuations in milk production during the lactation period relate to changes in CC [17].

In this study, BL linearly affects DMP, with the correlation coefficient being very weak (r=0.15), and it affects DMP as the determinant coefficient (R^2^) of 2.31%, the remaining 97.69% is influenced by other factors. The BL of the *Ruminantia* is correlated to the small intestine length, where a large portion of digestion and absorption of nutrients occurs. Those nutrients, among others, are processed into milk substituent in the mammary gland [18]. This fact explains that milk production will increase or decrease linearly, according to BL. A longer BL, followed by the length of the small intestine, is much more absorbent of nutrients, which could further increase the DMP.

### The effect of udder morphometry on DMP

The endocrine processes of mammogenesis and lactogenesis are genetic variables in milk production. The development of mammary glands occurs in a long-term process that requires reproductive hormones. In this study, the cows were in the lactation period, aged 4-8 years, with parity of 2-5, which means the morphometry of the udder was relatively stable and would not develop anymore.

Estrogen receptors are found in the mammary gland; therefore, estrogens have a direct impact on tissues. Meanwhile, an indirect effect of estrogens is the stimulation of prolactin (PRL) release from the pituitary and an increase in the number of PRL receptors in the mammary gland. Estrogen is also necessary for lactogenesis and causes the increased secretion of growth factors (insulin-like growth factor [IGF-1], and transforming growth factor-α) and the increased sensitivity of glandular cells. The critical factor for lactation initiation is an appropriate estrogen to progesterone ratio [19]. Changes in progesterone and PRL at the end of pregnancy lead to an early signal of lactation [20]. In lactating dairy cows, the PRL is released by the anterior pituitary gland as a response to stimulation at milking and suction [21].

Furthermore, PRL is very important to maintain lactation [22,23]. IGF-1 affects the maintenance of the healthy histological structure and the functioning of the mammary gland [24,25], and it plays a key role in cellular glucose metabolism, amino acid uptake, glycogen synthesis, mitogenesis, and lipogenesis [26], thus affecting milk yield mediated by nutritional status, as well as stimulating synthesis and secretion of milk [27]. Anatomically bigger udders of dairy cows mean more parenchymal tissue, including a higher number of alveoli as the primary milk producers, thus needing much more endocrine support. In this study, the value of the correlation coefficients (r) for FUH, RUH, and UC was 0.16 (very weak), 0.25 (weak), and 0.44 (moderate), respectively. These variables affected DMP (R^2^) as 2.63%, 6.16%, and 19.53%, respectively, while the remaining percentages were influenced by the other variables. These results were similar to those from the study of Zebu cows in the northern region of Cameroon, in which there were significant correlations between the diameters and heights of udders and milk yield [28].

The e-DMP of the dairy cows through the equation of linear regression in this study matched the r-DMP of the cows and corresponded to the DMP of dairy cows that were owned by smallholders in other areas. Lower FUH and UC (in the location of study II: Geger Village, District of Tulungagung) was followed by lower r-DMP, e-DMP-FUH, and e-DMP-UC. In contrast, if the other predictors in all of the locations of the study were similar, this was followed by similar r-DMP and e-DMP.

## Conclusion

The results from the present study indicate that DMP can be predicted using the body and udder morphometry based on the equations: −1.30+0.11*BL; 13.90+0.41* FUH; 11.02+0.18*RUH; and 3.87+0.16*UC. The best equation for e-DMP was based on UC.

## Authors’ Contributions

SS conceived the idea and designed the mainframe of this manuscript under the supervision of IM and PS. SS, TIR, and SM coordinated the field data collection. SS performed the statistical analysis and drafted the manuscript. IM, PS, and WW critically read and revised the manuscript for its intellectual content. SU contributed in coordinating the collection of field data with TIR and SM. All of the authors read and approved the final manuscript.
